# Progressive Dyslexia: Evidence from Hungarian and English

**DOI:** 10.3233/BEN-2012-119003

**Published:** 2012-03-19

**Authors:** Judit Druks, Jennifer Aydelott, Marios Genethliou, Helen Jacobs, Brendan Weekes

**Affiliations:** ^1^University College LondonLondonUK; ^2^Birkbeck CollegeLondonUK; ^3^University of Hong KongHong KongChina

**Keywords:** Models of reading, non-fluent progressive aphasia, bilingualism

## Abstract

We report a patient with non-fluent Primary Progressive Aphasia who was premorbidly literate in two alphabetic scripts, Hungarian (L1) and English (L2). Testing was performed over a two-year period to assess the impact of progressive illness on oral reading and repetition of single words. Results showed significant decline in oral reading in both languages, and an effect of language status in favour of oral reading in L1. Phonological complexity was a significant predictor of oral reading decline in both languages. Of interest, we observed an effect of language status on task performance whereby repetition was better in L2 than L1 but oral reading was better in L1 than L2. We conclude that language status has an effect on repetition and oral reading abilities for bilingual speakers with non-fluent Primary Progressive Aphasia.

## 1. Introduction

Studies of bilingual speakers who have acquired dyslexia in more than one language are an important source of data to test models of oral reading and written word recognition [13,21]. Take as an example studies reporting patients who produce cross-linguistic oral reading errors. Byng, Coltheart, Masterson, Prior and Ridoch [3] reported acquired dyslexia in a patient who premorbidly read in English and Nepalese – a syllabic Devanagari script with no orthographic similarity to English. Reading was better preserved in the second language English (L2) than the first language Devanagari (L1). Of interest, the patient could read aloud words written in Devanagari if he could respond in English (see also [11]). Raman and Weekes [16,22] reported a similar phenomenon in a Turkish (L1)/English (L2) speaker BRB who could produce the definitions of English printed homophones by responding in Turkish only (see also [2]).

Cross linguistic reading errors suggest that oral reading in bilingual acquired dyslexia is non-selective and language independent. However, language selective effects on oral reading in bilingual acquired dyslexia have also been reported. These effects tend to be seen in patients who premorbidly read in languages with different writing systems – alphabetic versus syllabic, alphabetic versus logographic and syllabic versus logographic (see [20,21] for a review). One such patient reported by Beland and Mimouni [2] displayed better reading of nonwords and more semantic errors in French (L2) than in Arabic (L1) but more translation errors in Arabic than French i.e. a response was produced in French when Arabic was tested. In Chinese-English speakers, Eng and Obler [7] observed more semantic reading errors in Chinese (L1) than English (L2). Kim, Na and Park [12] reported a bilingual dysgraphic patient who showed transposition errors between consonants and vowels when writing in English but not in Korean. Ohno, Takeda, Kato and Hirai [14] reported a Japanese-English patient who could read in English but could not read Kanji or Kana. Ibrahim [9] reported an Arabic-Hebrew speaker whose spontaneous writing was better in Hebrew than Arabic yet he could only spell single

ISSN 0953-4180/12/$27.50 © 2012 – IOS Press and the authors. All rights reserved words in Arabic. Ibrahim [10] reported the opposite pattern in patient MH (reading and writing in Arabic better than Hebrew). Ratnavalli et al. [17] reported two patients who had learned to read in English and Kannada (a Dravidian language of South India with a semi-syllabic script). For both patients the errors made when reading Kannada script (L1) were predominantly non-words while the errors made when reading in English (L2) were words. Karanth [11] reported a patient AG who produced semantic errors when reading and spelling in English (L2) co-incident with a total inability to read in Hindi (L1). Chengappa et al. [4] reported a patient who showed symptoms of surface dyslexia in English but not in Kannada or in Hindi.

Language selective effects in bilingual dyslexia suggest that oral reading in two different scripts requires separate reading systems that can be damaged independently at the cognitive and neural levels. However, language selective effects on oral reading in bilingual acquired dyslexia could be due to several different factors. For example, individual differences in premorbid proficiency, age of acquisition of the orthography or the premorbid familiarity with each language could explain dissociations in performance. Extant evidence comes from patients recovering from a brain injury. Therefore, variables such as individual differences in recovery of attention and memory could all impact on cognitive function. Because performance on oral reading tasks necessarily depends on these cognitive processes, data from all reported patients is difficult to interpret. These problems limit the potency of data for development of theoretical models of oral reading in bilingual speakers.

An alternative is to examine acquired dyslexia in bilingual patients with a progressive illness. Here we report the oral reading of a bilingual patient who had a progressive disorder: non-fluent primary progressive aphasia (nfPPA). According to the language non-selective view of acquired dyslexia in bilingual speakers, oral reading in both languages should deteriorate in a similar way. We tested this hypothesis with a patient JB who was pre-morbidly literate in two alphabetic scripts Hungarian and English prior to the onset of nfPPA affecting both languages.

## 2. Case description

JB was a 66 year-old right-handed man who was a proficient bilingual speaker premorbidly. JB's first language was Hungarian (L1). He received eight years of education in Hungary. He then migrated to England at the age of fourteen, where he married, and spoke English at home with his wife and children. His wife reported his pre-morbid use of English (L2) was excellent. Although he retained contact with Hungary and the Hungarian language, this was sporadic, and therefore it is safe to conclude that English was JB's dominant language. JB's wife reported more than five years of word finding problems and difficulties writing prior to seeking medical help. When he came to our attention, his connected speech was already very impaired. While he could only speak a few isolated words in English, his Hungarian was somewhat better. Word comprehension was initially intact, and JB was able to participate in everyday activities. However, at first testing JB's verbal output had declined markedly and speech production was extremely effortful. Comprehension was relatively well preserved although better in Hungarian than English. IQ was estimated to be in the normal range (verbal IQ of 83 and performance IQ of 99) with a significant decline after 1 year (verbal IQ of 61 and performance IQ of 74). In addition to language problems, JB demonstrated some impairment in calculation, digit span and praxis. However memory function, orientation in time and space, visuospatial abilities and face recognition were all preserved as observed in other cases of nfPPA [18]. Performance on tests of conceptual knowledge varied according to the grammatical class, with scores in the normal range for tests involving knowledge of objects (Pyramids and Palm Trees test) [8], but impaired for tests involving knowledge of actions (Kissing and Dancing) [1]. Two MRI scans found sulcal widening, more prominent in the left hemisphere (2001) followed by loss of volume and profound atrophy in the perisylvian region, mainly on the left hand side (2003). Temporal and posterior cortical structures were preserved at time of testing.

### 2.1. Language assessments

Language testing was carried out at two time points 2002–2003 (T1) and 2004 (T2) in English and Hungarian. When possible, testing was performed with the same lexical items in English and Hungarian using translation equivalents constructed by the first author and her colleagues.

As expected in a patient with nfPPA, performance on tests of phonological output in single word repetition showed evidence of significant decline over time in both languages. Performance was more accurate if targets were shorter and phonologically less complex.

When words contained clusters, performance in English was better when the cluster was word-initial, and in Hungarian when it was word final. Phoneme addition errors became more common over time. Substitutions at T1 were typically of consonants, though vowel substitutions increased in frequency, so that by T2, both segment types were substituted with equal likelihood. Most consonant substitutions involved a different place of articulation to the target, and most vowel substitutions were schwa insertions. Changes in vowel duration were also common. This poses more of a problem in spoken Hungarian, because vowel length in Hungarian is phonologically contrastive and therefore has a greater effect on intelligibility than in English. It was noted that phonological clusters were usually simplified rather than substituted in Hungarian and English suggesting problems with assembly for articulation, rather than access to correct phonological representations for spoken output. In sum word length, frequency and phonological complexity were all significant factors affecting JB's accuracy in speech production in both Hungarian and English.

## 3. Experimental investigations

We expected deterioration to speech production and oral reading in both languages to follow a similar pattern. Therefore, we compared repetition and oral reading across two time points (T1 and T2) separated by 12 months. We also examined the effects of psycholinguistic variables on repetition and reading performance in each language. Hungarian has a transparent orthography with few irregularities in spelling to sound correspondences. By contrast, spelling to sound correspondences in English are more opaque with several irregularly spelled words e.g. yacht. Several consonants, clusters of consonants that correspond to a single phoneme (cs, sz, zs, gy, ly, ny, ty) and vowels, including long vowels (á, é, í, ó, ö, ő, ü, ű) are unique to Hungarian, whereas other written symbols (q, w, x, y) are all unique to English. The transparency of Hungarian orthography could in principle allow all words to be pronounced without lexical knowledge via reference to grapheme to phoneme rules, which is not true in English. Further, because Hungarian is an agglutinating language, words tend to be longer on average than their translation equivalents in English. Differences between languages in oral reading performance would therefore most likely be observed in effects of regularity and word length on production.

### *3.1. Materials and procedure*

JB's repetition and oral reading were examined with single words presented in English and Hungarian. For English there were 350 words organized into 7 lists, and in Hungarian there were 487 words organized into 6 lists. Each list contained one syllable, two syllable and three syllable words and each word length category contained phonologically simple (without consonant clusters) and phonologically complex (with consonant clusters at word initial, mid-word and word-final positions). Stimuli were repeated 16 months apart (T1 and T2). Performance was recorded and transcribed for later analysis.

### *3.2. Results*

JB's performance in reading and repetition at T1 and T2 in English and Hungarian are summarized in Table 1. Performance deteriorated (halved) over time in each language. JB performed significantly worse at T2 than T1 in reading $\chi^2(1) = 45.22$, $p < 0.001$ and in repetition $\chi^2(1) = 64.92$, $p < 0.00$. Comparison of scores on reading and repetition tasks showed that at T1, repetition was significantly better than reading in English $\chi^2(1) = 11.47$, $p = 0.001$ but not in Hungarian, where the opposite pattern was observed i.e. reading was better than repetition. However, at T2, performance was poor in both tasks and differences were no longer significant $\chi^2(1) = 1.16$, $p = 0.282$. Better performance in repetition than oral reading in patients with nfPPA was observed by Croot et al. [5] who interpreted the effect as due to more direct activation of phonological representations for repetition than for oral reading. Comparing across languages, there was a significant difference in repetition scores at T1, with repetition in English better in Hungarian $\chi^2(1) = 5.11, p = 0.024$. The opposite was true of oral reading scores, Hungarian being better than English $\chi^2(1) = 4.51, p = 0.03$. At T2, there was no significant difference in repetition across language $\chi^2(1) = 1.66$, $p = 0.19$ but a significant difference between the languages for oral reading $\chi^2(1) = 3.563, p = 0.05$ with better oral reading in Hungarian. Thus, although repetition in English was superior at T1, repetition in English deteriorated faster than in Hungarian. Within English, repetition scores were significantly higher than reading scores at Time 1 $\chi^2(1) = 11.47, p = 0.00$ however the difference between tasks was not significant at Time 2 $\chi^2(1) = 1.16$, $p = 0.28$. When these scores were summed to compare total reading score with total repetition score

Table 1
Number (%) of words repeated and read aloud at T1 and T2 in English and in Hungarian

| | Time 1 | | Time 2 | |
|---|---|---|---|---|
| | English | Hungarian | English | Hungarian |
| Reading | 112/350 (32) | 98/242 (40.5) | 49/350 (14) | 62/319 (19.44) |
| Repetition | 149/350 (42.57) | 80/240 (33.33) | 59/350 (16.86) | 65/311 (21) |

Table 2
Regression coefficients at T1 and T2 for repetition in English and in Hungarian

| | Co-efficient | Standard error | Degrees of freedom | Significance value |
|---|---|---|---|---|
| **English T1** | | | | |
| Number of syllables | 7.640 | 15.370 | 1 | $p = 0.619$ |
| Number of phonemes | −0.330 | 0.168 | 1 | $p = 0.049$ |
| Constant | −6.241 | 15.401 | 1 | $p = 0.685$ |
| **English T2** | | | | |
| Imageability | 0.004 | 0.002 | 1 | $p = 0.068$ |
| Number of phonemes | −1.195 | 0.242 | 1 | $p < 0.001$ |
| Constant | 1.025 | 1.259 | 1 | $p = 0.416$ |

| | Wald statistic | Degrees of freedom | Significance value |
|---|---|---|---|
| **Hungarian T1** | | | |
| Number of syllables | 20.367 | 3 | $p < 0.001$ |
| Constant | 0.154 | 1 | $p = 0.694$ |
| **Hungarian T2** | | | |
| Number of syllables | 21.185 | 3 | $p < 0.001$ |
| Constant | 0.06 | 1 | $p = 0.807$ |

(across time), repetition performance was significantly better than oral reading $\chi^2(1) = 11.56$, $p < 0.001$. Within Hungarian, repetition scores were no different to oral reading scores at Time 1 $\chi^2(1) = 1.21$, $p = 0.29$ or Time 2 $\chi^2(1) = 0.07$, $p = 0.79$. In sum, the results show a similar pattern of deterioration on each task in both languages, as expected on the language non-selective view.

We investigated factors that might explain the interaction between language type and task. To identify the factors that affect repetition and oral reading performance in English and Hungarian, logistic regression analyses were performed. The primary variable of interest was the phonological complexity of words in each language given the pathology of the patient and differences across languages in average word length. Phonological complexity was defined as the number of consonant clusters, phonemes, letters and syllables. Backward multiple logistic regression was used to ensure that those variables making no significant contribution to the regression equation were eliminated at each stage of the analysis. Variables were entered into the initial stage of the model and then removed until only significant contributors remained.

Results for repetition in Hungarian found similar effects on T1 and T2 performance. Coefficients and associated probability values are summarised in Table 2. At T1, the model was significant $\chi^2 = 33.94$, $p < 0.001$ classifying 66.7% of items and number of syllables was the best predictor. At T2, the model was significant $\chi^2 = 62.242$, $p < 0.001$ classifying 79.2% of items and number of syllables was the best predictor. Results for repetition in English found very similar effects. Coefficients and associated probability values are summarised in Table 2. At T1, the model was significant $\chi^2 = 35.95$, $p < 0.001$ classifying 62.6% of items and number of syllables and phonemes were the best predictors. At T2, the model was significant $\chi^2 = 44.33, p < 0.001$ classifying 80.5% of items with number of phonemes the best predictor.

Results for oral reading in Hungarian found similar effects on T1 and T2 performance. Coefficients and associated probability values are shown in Table 3. At T1, a model containing number of consonant clusters and number of syllables was significant $\chi^2 = 42.54$, $p < 0.001$, and correctly classified 68.6% of items. Removing phoneme and letter numbers made no difference to the predictive power of the model. At T2, a model containing syllable number significantly predicted accuracy $\chi^2 = 69.453, p < 0.001$ and correctly classified 80.6% of items. Removing number of consonant clusters, phonemes and letters made no difference to the predictive power of the model. Results for reading in English found similar patterns. Coefficients and proba-

Table 3
Regression coefficients at T1 and T2 for oral reading in English and in Hungarian

| | Co-efficient | Standard error | Degrees of freedom | Significance value |
|---|---|---|---|---|
| **English T1** | | | | |
| English written word frequency | 0.729 | 0.292 | 1 | $p = 0.012$ |
| English number of letters | −0.791 | 0.137 | 1 | $p < 0.001$ |
| Constant | 2.051 | 0.807 | 1 | $P = 0.011$ |
| **English T2** | | | | |
| English written word frequency | 1.377 | 0.673 | 1 | $p = 0.041$ |
| English number of letters | −0.836 | 0.190 | 1 | $p < 0.001$ |
| Constant | 1.224 | 1.090 | 1 | $p = 0.262$ |

| | Wald statistic | Degrees of freedom | Significance value |
|---|---|---|---|
| **Hungarian T1** | | | |
| Complexity | 12.195 | 2 | $p = 0.002$ |
| Number of syllables | 23.422 | 3 | $p < 0.001$ |
| Constant | 2.278 | 1 | $p = 0.131$ |
| **Hungarian T2** | | | |
| Number of syllables | 20.120 | 3 | $p < 0.001$ |
| Constant | 0.148 | 1 | $p = 0.701$ |

bility values are shown in Table 3. At T1, a model with written number of letters was significant $\chi^2 = 52.24$, $p < 0.001$, and correctly classified 66.0% of items as correct or incorrect. At T2, a model with number of letters predicted reading accuracy $\chi^2 = 32.64$, $p < 0.001$, and correctly classified 82.8% of items. Removing number of consonant clusters, phonemes and syllables made no difference to the predictive power of any model. In sum, word length (the number of syllables, the number of phonemes and the number of letters) was a significant predictor of repetition and oral reading success in both languages across time. This effect was manifest in the number of syllables in Hungarian (and not English) and number of letters in English (and not Hungarian).

## 4. Discussion

The results reveal two novel effects on oral reading performance in a bilingual patient with a progressive illness: a main effect of language status with reading better in Hungarian (L1) than English (L2) and a main effect of time with oral reading more impaired at T2 compared with T1. There was no interaction between variables. Word length explained both repetition and oral reading performance in each language across time. These results are therefore compatible with the language non-selective view of acquired dyslexia in bilingual speakers articulated above.

According to the contrasting language selective view of bilingual language processing, differential effects of language type on the deterioration of language performance across time might be expected. For example a patient with impaired phonological production due to nfPPA might have more difficulty producing phonologically complex words in a language with longer words. There was evidence to support this view. For example, repetition was more difficult for JB in Hungarian than in English. However we also found that word length had a pervasive and significant effect on oral repetition and also on oral reading in both languages so phonological complexity does not appear to be more of a constraint on one language or task than any other.

The data showed that although reading was better preserved in L1, deterioration to oral reading performance was similar in L1 and L2. The results do show an effect of language, with worse performance in L2 than L1 the only exception being better oral repetition in L2. Effects of language could be due to differences in language *type* or differences in language *status*. Effects of language type in the present case refer to linguistic distinctions between Hungarian which is more agglutinating, phonologically complex and contains more transparent mappings between orthography and phonology compared to English. However, a more likely explanation of the effect of language on JB's performance is differences in language status (L1 versus L2). We therefore contend that differences in performance between languages in bilingual dyslexia are due to effects of language status and not effects of language type (for a similar view [22]).

Effects of language status may reflect strength of connections between representations of words in L1 and L2. All models of bilingual language processing assume that the weights on nodes in a fully integrated lexicon are strongest for frequently encountered words and these are less vulnerable to brain damage. According to all cognitive models with an integrated lexicon, brain damage is less likely to have an effect on language processing in L1 than L2 because of (typically) greater exposure to words in the first acquired language than the second acquired language. Note however that the assumption of greater association strength between weights in a computational model of bilingual language processing allows for factors such as language dominance (familiarity) – as well as age of acquisition – to have an impact on task performance.

We note from the case report that JB acquired English at the age of 15 years but was able to read in Hungarian from an early age. He thus learned to read English many years after he began to read Hungarian. Therefore, JB may have enjoyed more exposure to written words in Hungarian than English in his premordid reading experience. However, although Hungarian was JB's first acquired language, and the language in which he learnt to read, it was not his most familiar or dominant language. JB had communicated in English almost exclusively for over fifty years. Therefore although first exposure to Hungarian words was earlier than English, L1 was not the most frequently encountered language throughout JB's life. The point we wish to emphasise from these findings is that it is not necessarily the age of acquisition or language dominance that will predict the patterns of language deterioration observed in bilingual nfPPA. Instead, it seems that pre-morbid exposure to a language serves to protect some language skills from initial deterioration in this progressive illness. Pre-morbid exposure will of course be very different across spoken and printed modalities and thus the patterns of language deterioration in nfPPA will be highly variable even within an individual patient depending on many factors such as the frequency of language exposure at home or school, work place, social networks and activities conducted in one language compared to another e.g. crosswords or listening to music.

Cognitive models of bilingual oral reading such as the Bilingual Interactive Activation BIA+ model proposed by Dijkstra and colleagues [6,19] do not distinguish between age of acquisition and language dominance to explain reported effects of language status on written word recognition and reading in bilingual speakers who do not have brain damage. Moreover, it is possible at least in principle, for the second acquired language to dominate the connection weights between orthography and phonology within an integrated lexicon if exposure to L2 is more frequent overall across the lifespan. What JB's data reveal is that the age of acquisition of a language is no better than familiarity or dominance in predicting deterioration to oral reading in bilingual progressive dyslexia. Instead it seems that stimulus length predicts performance as reported in studies of monolingual speakers with a progressive illness including nfPPA [5,18].

Phonological complexity i.e. whether a consonant cluster was present in Hungarian and word length i.e. syllable and phoneme number in Hungarian and number of letters in English predicted JB's oral repetition and oral reading. The BIA+ model can explain these effects on oral reading as a consequence of reliance on the sublexical reading pathway, possibly due to damage in the (unimplemented) semantic reading pathway. Reliance on a common sublexical reading pathway manifests differently across language. In Hungarian, the number of consonant clusters and the number of syllables reveals operation of the sublexical pathway whereas in English it is number of letters. This does not imply that other measures (number of phonemes) are not also significant predictors of progressive dyslexia in nfPPA. Measures of phonological complexity are necessarily correlated. To disentangle correlated effects, it will be necessary to conduct analyses in which, for example, one complexity variable is held constant while another is varied i.e. testing words of one syllable differing in number of phonemes. It is premature to propose a measure of word length that is critical given problems of multi-collinearity. Further analysis would unlikely alter our conclusion that phonological complexity predicts oral reading performance in progressive dyslexia although this hypothesis clearly requires more research.

Most computational models of reading are language specific (developed for English, Chinese and German). These models share assumptions based on learning via strengthening the connections between information nodes that represent unique features of the orthographic and phonological units in a language. However, there is no requirement that computational models be language specific. Plaut [15] showed that differences in script could be instantiated in a PDP model by adjusting weights to reflect consistency in the mappings between orthography and phonology. In a transparent script such as Hungarian the weightings between orthography and phonology are likely to be stronger than the mappings between orthography and phonology in English, reflecting the reliability of repeated associations between print and sound. In English, the division of labour for oral reading is more likely to be dependent on the mappings between orthography, semantics and phonology allowing reading of irregularly spelled words. Like all PDP models, the BIA+ model is a framework that processes written words on the basis of similarity to representations in an integrated lexicon. It is thus independent of within language constraints. The critical assumption of the BIA+ model is that oral reading in a bi-script reader activates a common set of orthographic units regardless of type of script. Our results support this assumption if the two languages use a common orthography. It is interesting to speculate whether computational models of oral reading should continue to be language specific models.

## 5. Conclusion

This is the first report on repetition and oral reading of a bilingual speaker with progressive dyslexia. We contend that the decline of oral reading ability in bilingual patients with nfPPA depends on the phonological complexity of the word across all languages tested. Oral reading also depends on the familiarity of the patient with the pre-morbid language. These effects could be simulated in computational models of bilingual reading such as the BIA+. Remarkably and rather unexpectedly, the pattern of language exposure to a language across the lifespan seems to mitigate somewhat against language decline even if L2 is a relatively late acquired language.
